# Hematopoietic stem cell transplantation dilemma during the COVID-19 era

**DOI:** 10.2217/fon-2020-0414

**Published:** 2020-05-27

**Authors:** Ramy Samaha, Joseph Kattan

**Affiliations:** ^1^Hotel-Dieu de France University Hospital, Faculty of Medicine, Saint Joseph University, Beirut, Lebanon

**Keywords:** coronavirus, COVID-19, hematology, hematopoietic stem cell transplantation, oncologist, oncology

Toward the end of 2019, WHO started taking notice of a new highly contagious virus, the novel coronavirus (2019-nCoV), originating in the city of Wuhan in Hubei province, China [1]. Since then, the virus now known as severe acute respiratory syndrome coronavirus 2 (SARS-Cov-2) has been declared a pandemic by the WHO with more than 2 million coronavirus cases and claiming the lives of more than 160,000 persons [2–4]. SARS-Cov-2 incubation period ranges from 1 to 14 days with a median of 5 days [5]. Droplets and close contact are the main routes of transmission, but reports suggest that transmission can occur through aerosols [6]. Presentation varies from no or mild symptoms such as fever and dry cough, to more severe symptoms such as pneumonia and acute respiratory distress syndrome [7]. A series of measures has been implemented by countries, following CDC and WHO recommendations, to limit disease spread. These measures include, but are not limited to, social distancing, frequent hand wash, high alert for suspicious symptoms [8,9]. The pandemic has proved to be stressful and deleterious on healthcare and the economic system both on the short- and long term [10,11]. No vaccine has been found to date, and although many therapeutic options such as hydroxychloroquine and remdesivir are under investigation, supportive and symptomatic care remain the treatment of choice [12,13].

## COVID-19 & cancer

GLOBOCAN 2018 expects cancer to be the leading cause of death in every country in the 21st century, estimating 18.1 million new cancer cases and 9.6 million cancer deaths [14]. Cancer patients are at higher risk of any infection due to the immunosuppression frequently caused by cancer itself and cancer related treatment [15–17]. Growing evidence support the fact that cancer patients are at higher risk of COVID-19 infection and related complications including death [18,19]. Subsequently, oncologists and oncology societies have been forced to apply some practice changes when possible such as: delay or omission of treatment, switching from intravenous to oral medication, using neoadjuvant chemotherapy as a bridge to delay surgery, prescribing marginally less effective treatment but with lower risk of immunosuppression and increasing use of white blood cell growth factors [20,21].

## Hematopoietic stem cell transplantation

Initially described in the 1950s on mice models, hematopoietic stem cell transplantation (HSCT) was first used on humans in 1957 [22,23]. This accomplishment and the continued research on bone marrow transplant earned Dr E Donnall Thomas the Nobel Prize of physiology and medicine in 1990 [24]. Since, allogeneic and autologous bone marrow transplantation have revolutionized the treatment for a variety of malignant hematologic and solid tumors as well as nonmalignant hematologic and immunologic diseases [25]. Bone marrow transplant starts with mobilization then harvesting and cryopreservation of hematopoietic stem cells from histocompatible related or unrelated donors in allogenic, and from the patient’s bone marrow or peripheral cells in autologous transplantation. Thereafter comes the conditioning phase to eradicate malignant and host immune cells using various myeloablative conditioning (MAC), non myeloablative, and reduced intensity conditioning regimens. MAC regimens offer lower risks of relapses on the expense of higher toxicity, while non myeloablative regimens offer a better safety profile in a specific population with high comorbidities but with a higher rate of relapse [26,27]. These regimens are based on various intensity of chemotherapy and/or radiation therapy [25,28]. Allogenic HSCT offers the benefit of graft versus tumor effect able to eradicate malignant cells after nonablative conditioning regimens [29]. Lastly comes the transfusion of stem cells and homing to the recipient’s hematopoietic microenvironment with engraftment in the bone marrow niches [30]. The goal of HSCT is curative, although cure rates in high-risk malignancies is substantially lower than patients’ expectations [31].

## COVID-19 & HSCT

Many challenges and constraints face patients undergoing HSCT and their informal caregivers with a long and unpredictable illness trajectory [32]. Allogenic HSCT recipients frequently need unscheduled hospital admissions and should temporarily relocate to stay close to the hospital [33,34]. After conditioning patients lose all T- and B-lymphocytes, losing all immune memory accumulated through the years in addition to damaged mucocutaneous barriers. Thus, HSCT are a highly susceptible population to opportunistic infections [35]. Community viral respiratory infection can occur in about half of HSCT recipients, and infectious complications have been reported to be as high as 92% [36,37]. Therefore, CDC recommends strict adherence to infection control measures such as wearing protective gear and isolation, as well prophylactic antimicrobial and antifungal regimens [35]. Evidently, respiratory viral upper tract infection is less common in non-MAC regimens compared with myeloablative ones during the first 100 days, although rate of acquisition beyond the 100 days was similar [38]. Gil *et al.* further showed a very high-risk of infection (92.3% of patients) after autologous HSCT using high-dose conditioning regimens [37]. In addition, in 2015, Campbell *et al.* showed the importance of pretransplant viral status and demonstrated a statistically significant lower overall survival in the group diagnosed with respiratory virus (84.5%) compared with the uninfected group (92.1%) on day 100. These results justified viral panels in symptomatic patients as well as delaying transplant in symptomatic patients [39]. Furthermore, allogenic HSCT recipients have poor outcome after ICU admissions nevertheless Lengliné *et al.* showed a recent improvement of outcomes in ICU with a 3-month mortality of 70% after mechanical ventilation with infection being the main cause of death (30.6%) [40].

Although the goal of HSCT is curative and patient might suffer harm if the procedure is delayed, treatment related toxicities and technical difficulties will be amplified and overwhelming during this pandemic. Thus, hematologic societies, such as EBMT and ASTCT, have been quick to release new interim guidelines to help physicians during this very particular time of viral outbreak. First of all, before the start of the procedure, the availability of adequately trained staff, facilities including ICU beds and ventilators should be checked [41]. Next is assuring the availability of the cell therapy product through minimum disruption of couriers delivering, considering travel restrictions and strained healthcare system, as well as cryopreservation products as early as possible before conditioning and having alternate donor options [41,42]. Furthermore, all patients should be tested SARS-Cov2 PCR test before the start of the procedure [41,43]. As to donors, SARS-CoV-2 can also be detected in blood, but screening for SARS-CoV-2 in blood products is not recommended by American Association of Blood Banks guidelines and considering risk factors for infection and exposure history during the last 28 days is recommended by the US FDA, although there has been no reported transmission by blood products [44–46]. Different scenarios are presented for recipient and donor, although clinical judgment of the treating physician remains the most important factor. These clinical scenarios are as presented by hematologic societies are summarized in Table 1 [41,43].

**Table 1.** Recommendation for COVID-19 and hematopoietic stem cell transplantation (ASTCT and EBMT).

	ASTCT	EBMT
**HSCT recipient**		
SARS-Cov2-positive	HSCT deferred until patient is asymptomatic and two negative PCR tests are obtained at least 1 week apartConsider minimum intensity conditioning regimen	Low risk of disease: deferred for 3 months
		High-risk of disease: deferred until patient is asymptomatic and two negative PCR at least 24 h apart
Close contact with SARS-Cov2-positive person or travel to high-risk area according to CDC or close contact with a person who has traveled to high-risk area	Deferred for 14–21 days of last contact, or two negative PCR tests are obtained at least 1 week apartClose monitoring for symptoms	Low risk of disease: deferred for 14–21 days of last contact
		High-risk of disease: deferred for 14–21 days of last contact according to clinical judgment
		A negative PCR must be confirmed before proceeding
High prevalence of COVID-19 in community	SARS-Cov2 PCR at initial evaluation and 2 days before conditioning	
	Consider interim treatment or longer deferral when possible	
**Donor**		
SARS-Cov2-positive	Donor is ineligibleWill be eligible again if asymptomatic after 28 days and a negative PCR	Donor is excluded and duration is unclear
Close contact with SARS-Cov2-positive person or travel to high-risk area according to CDC or close contact with a person who has traveled to high-risk area	Excluded for 28 days	
	In high-risk situation donor may be considered eligible if asymptomatic with a negative PCR. Close follow-up	Excluded for 28 daysException is made if bone marrow transplant is urgent and donor asymptomatic with a negative PCR and no other eligible donors: earlier collection is possible

HSCT: Hematopoietic stem cell transplantation.

## Conclusion & future perspective

With the number of COVID-19 cases rising every day, the management of cancer patients is becoming more challenging. HSCT recipients represent a vulnerable population due to the frequent severity of their baseline hematologic malignancies and the high risk of complications, notably infectious complications, after treatment initiation. However, bone marrow transplant remains the only curative therapy in many of these malignancies, and time is crucial for these patients. Hence, hematologic society’s guidelines have come to help clinical judgment during this pandemic. “*Primum non nocere”* remains the pillar of medical practice and decision making for physician and many questions are left to be answered: How long is delaying HSCT safe? How far could the curative effect of HSCT be maintained? Real-world data of HSCT patients from the COVID-19 affected areas are eagerly needed to shed light on many of these questions and help clinicians make optimal judgments for their patient.
